# Targeting MUC16 to Reverse Anoikis Resistance: A Promising Strategy for Metastatic Lung Adenocarcinoma Therapy

**DOI:** 10.7150/jca.131494

**Published:** 2026-03-30

**Authors:** Kulsoom, Wajahat Ali, Zhe Ma, Wen Cai, Zhenlong Wang, Fu Wang

**Affiliations:** 1 Department of Urology, The Second Affiliated Hospital of Xi'an Jiaotong University, Xi'an, Shaanxi 710004, China.; 2 School of Pharmacy, Shaanxi Institute of International Trade & Commerce, Xianyang 712046, Shaanxi, China.; 3 School of Basic Medical Sciences, Xi'an Jiaotong University, Xi'an 710061, China.

**Keywords:** *MUC16*, Anoikis resistance, LUAD, FAK/PI3K-AKT signaling, Therapeutic targeting

## Abstract

**Background:**

Although the mucin glycoprotein *MUC16* is well established as an oncogenic biomarker in ovarian cancer, its mechanistic contribution to lung adenocarcinoma (LUAD) remains poorly defined. This study integrates multi-omics profiling and functional validation to uncover *MUC16* as a novel determinant of detachment-induced survival in LUAD.

**Methods:**

Transcriptomic and clinical data from TCGA-LUAD and GEO cohorts were analyzed to identify differentially expressed anoikis-related genes. Functional enrichment, GSVA, and network analyses delineated key signaling pathways. Multi-omic integration including copy-number, methylation, immune infiltration, and pharmacogenomic data was used to explore upstream regulatory mechanisms. Experimental assays were performed in A549 cells following siRNA-mediated *MUC16* silencing, assessed by qRT-PCR, wound-healing, and transwell migration analyses.

**Results:**

Nineteen overlapping anoikis-related differentially expressed genes (ARDEGs) were identified, with *MUC16* displaying the most significant upregulation in LUAD and a strong association with poor overall survival (HR = 1.04, p < 0.001). Pathway enrichment indicated activation of cell-adhesion, Hippo, and PI3K-AKT signaling networks. Multi-omic analysis revealed that promoter hypomethylation and copy-number gain drive *MUC16* overexpression, which correlates with reduced cytotoxic T-cell infiltration and an immunosuppressive microenvironment. Functionally, *MUC16* knockdown diminished cell adhesion, migration, and wound closure, consistent with loss of detachment survival capacity.

**Conclusion:**

This work establishes *MUC16* as a mechanistic mediator of metastatic competence in LUAD, acting through adhesion-linked PI3K-AKT signaling to protect tumor cells from detachment-induced apoptosis. Therapeutic inhibition of MUC16 may restore apoptotic susceptibility and suppress metastatic spread in lung cancer.

## 1. Introduction

Lung cancer remains the most lethal malignancy worldwide, accounting for over 1.8 million deaths annually, with survival rates for advanced-stage disease stagnating below 20% despite recent advances in targeted therapy and immunotherapy 1-3. Metastatic dissemination is the leading cause of mortality in lung cancer; however, the molecular mechanisms that enable circulating tumor cells to survive detachment and colonize distant organs remain incompletely understood 4-6.

Anoikis, is a specialized form of programmed cell death triggered by loss of attachment to the extracellular matrix (ECM) 7-9. Under physiological conditions, anoikis prevents the aberrant survival of detached epithelial cells, thereby maintaining tissue homeostasis. In contrast, cancer cells frequently acquire anoikis resistance, which allows them to survive during intravasation, circulation, and subsequent colonization of secondary sites 8, 10, 11. Emerging evidence has identified anoikis resistance as a hallmark of lung cancer aggressiveness, sustained through activation of signaling cascades such as FAK-Src, PI3K-AKT, NF-κB, and epithelial-mesenchymal transition (EMT) regulators 12-14. Although recent transcriptomic studies have delineated anoikis-related gene signatures that stratify patient prognosis and reveal immune microenvironmental interactions in lung adenocarcinoma (LUAD), these findings remain largely computational, with limited experimental validation 15-17.

Among the putative regulators of anoikis, the mucin family glycoprotein *MUC16* (also known as *CA125*) has gained increasing attention. Well established as a clinical biomarker in ovarian carcinoma, *MUC16* is aberrantly expressed in lung cancer, where it has been implicated in promoting cell proliferation, migration, and immune evasion 18-20. However, its potential role in regulating anoikis resistance and metastatic progression in lung cancer has not been elucidated. This knowledge gap is of particular significance because *MUC16* has recently emerged as a promising therapeutic target, with antibody-drug conjugates and immune-based therapies currently under development 21-23.

**Figure 1:**
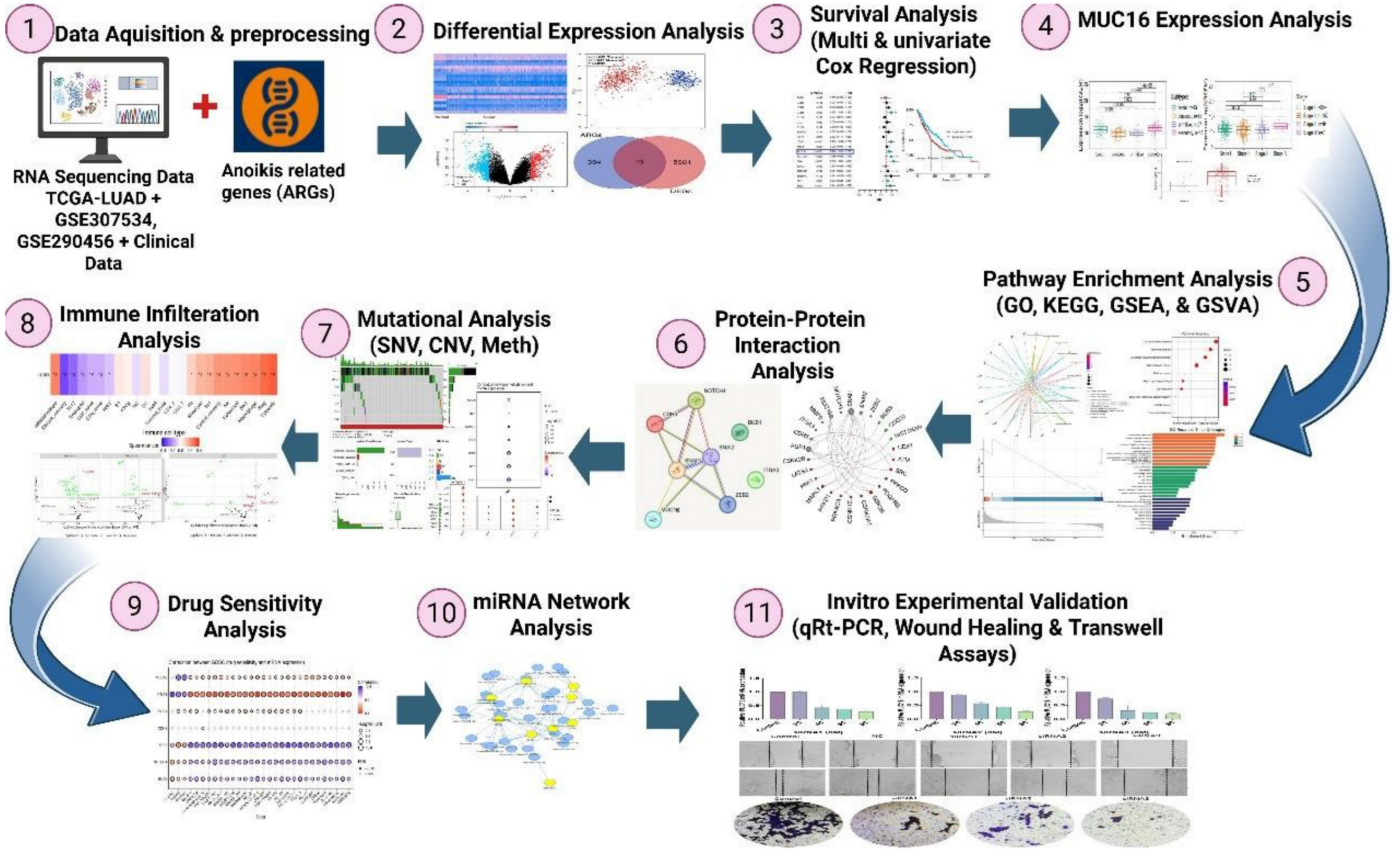
Scheme of the present study design.

Given that anoikis resistance is a defining feature of metastatic competence and that the role of *MUC16* in this process remains unexplored in lung cancer, the present study provides the cutting-edge comprehensive evidence linking *MUC16* expression to anoikis evasion in LUAD. To elucidate this relationship, we performed integrative transcriptomic analyses using TCGA and GEO datasets to identify differentially expressed anoikis-related genes (ARGs) and construct a prognostic signature (**Figure 1**). Multi-omic integration and immune deconvolution further characterized MUC16's regulatory landscape and tumor microenvironment associations. Finally, siRNA-mediated knockdown of *MUC16* in A549 cells was conducted to functionally validate its role in adhesion, migration, and anoikis resistance. Collectively, our study establishes a clinically and mechanistically grounded framework that connects anoikis biology to lung cancer progression and identifies *MUC16* as a novel biomarker and potential therapeutic target in lung adenocarcinoma.

## 2. Methodology

### 2.1. Data acquisition and preprocessing

Transcriptomic and corresponding clinical data for lung adenocarcinoma were obtained from the TCGA-LUAD cohort, comprising 535 tumor samples and 59 adjacent normal lung samples after quality control. In addition, two independent GEO datasets were included for validation: GSE31210 (226 LUAD samples) and GSE50081 (181 LUAD samples). Only samples with complete expression profiles and relevant clinical annotations were retained for downstream analyses.

The data cutoff date for all downloads was June 2024 to ensure the inclusion of the most updated cases. Raw expression profiles (FPKM format) were converted to Transcripts Per Million (TPM) to enable inter-sample comparability, followed by log₂(TPM + 1) transformation. Batch effects across different datasets were corrected using the ComBat algorithm from the *sva* R package (version 4.2.2). Only protein-coding genes present in >80% of samples were retained for downstream analyses. Clinical metadata, including survival status, stage, age, and gender, were curated and harmonized with the expression matrix. A curated list of anoikis-related genes (ARGs) was retrieved from the GeneCards, HADb, and MSigDB databases using “anoikis” as the keyword. Genes with a relevance score > 2 in GeneCards and annotated in GO biological processes related to cell detachment, apoptosis, and adhesion were included.

Differential expression analysis was performed using the “limma” package in R (version 4.2.2), applying |log₂FoldChange| > 1 and adjusted p < 0.05 (Benjamini-Hochberg). Volcano plots, Principle component analysis (PCA), volcano plot and heatmaps were generated using the “ggplot2” and “pheatmap” packages. Figure 1 shows the detailed flow chart of the study.

### 2.2. Prognostic and Survival Analysis

Univariate and multivariate Cox proportional hazards models were constructed using the “survival” and “survminer” R packages. Variables included MUC16 expression, age, sex, stage, and tumor purity. Kaplan-Meier curves were drawn for high- vs low-expression groups (median cutoff). Model validity was confirmed through the likelihood ratio, Wald, and log-rank tests. Tumor purity was included as a covariate in multivariate Cox regression analyses to account for its potential impact on bulk RNA-seq-based gene expression quantification and immune infiltration estimation. Variations in tumor cellularity can influence measured transcript abundance and confound associations between gene expression, immune contexture, and clinical outcome. Tumor purity estimates were obtained from TCGA annotations and incorporated to ensure robust and unbiased survival modeling.

### 2.3. Functional Enrichment Analysis (GO, KEGG, and GSEA)

Gene Ontology (GO) and Kyoto Encyclopedia of Genes and Genomes (KEGG) pathway analyses were performed using the “clusterProfiler”, “enrichplot”, and “org.Hs.eg.db” R packages. Biological process (BP), cellular component (CC), and molecular function (MF) categories were analyzed with a significance threshold of FDR < 0.05. Gene Set Enrichment Analysis (GSEA) was conducted using the “fgsea” package with 10,000 permutations and the MSigDB *C2 and C5* gene sets to identify enrichment of adhesion-, cytoskeleton-, and apoptosis-related pathways between *MUC16*^high and *MUC16*^low samples.

### 2.4. Gene Set Variation Analysis (GSVA)

To assess pathway activity variation, GSVA was performed using the “GSVA” R package (version 1.42.0). Hallmark gene sets (h.all.v7.5.1.symbols.gmt) were used as the reference. GSVA enrichment scores were compared between *MUC16*-silenced and control groups using the “limma” package, and significant pathways were visualized as enrichment heatmaps.

### 2.5. Protein-Protein Interaction and Network Construction

Protein-protein interaction (PPI) networks were generated using the STRING v11.5 database (confidence > 0.7). Network visualization and hub-gene analysis were performed in Cytoscape v3.9.1 using MCODE and cytoHubba plug-ins. Functional modules were annotated using the “ClueGO” plug-in to highlight enrichment in EMT, adhesion, and cell-cycle regulation pathways.

### 2.6. Multi-Omic Characterization

Copy-number variation (CNV) and single-nucleotide variant (SNV) data for LUAD were retrieved from cBioPortal (https://www.cbioportal.org) and GDC Portal. Somatic copy-number alteration (SCNA) segments were processed using GISTIC 2.0.

Methylation β-values were obtained from the TCGA Illumina 450 K platform. Spearman's correlation between promoter methylation and *MUC16* mRNA expression was computed to assess epigenetic regulation. Promoter-associated CpG probes were defined based on Illumina 450K annotations, including TSS200, TSS1500, 5′UTR, and first exon regions (±2 kb from the transcription start site).

### 2.7. Immune Infiltration Analysis

Tumor immune infiltration was estimated using CIBERSORT, TIMER2.0, and xCell algorithms. Normalized TPM data were deconvoluted to quantify 22 immune-cell types. Associations between *MUC16* expression (or mutation/amplification status) and immune-cell infiltration were computed via Spearman correlation. Results were visualized as correlation heatmaps and grouped bar plots using the “ComplexHeatmap” package.

### 2.8. Pharmacogenomic Correlation Analysis

Drug-sensitivity data were obtained from the Genomics of Drug Sensitivity in Cancer (GDSC) database. Spearman correlation between *MUC16* expression and half-maximal inhibitory concentration (IC₅₀) values for > 200 agents was computed using the “pRRophetic” R package. Drugs showing |r| > 0.3 and FDR < 0.05 were considered significantly correlated.

### 2.9. miRNA-mRNA Regulatory Network Construction

miRNAs targeting *MUC16* and its co-expressed genes (*NOTCH4, SNAI1/2, ITGA3, BUB1*) were predicted using miRDB, TargetScan, and miRTarBase. Common miRNAs were used to build an interaction network in Cytoscape, and functional annotation was performed via the miEAA 2.0 web tool to highlight EMT- and integrin-related regulatory clusters.

### 2.10. Cell Culture

A549 cells were cultured in Dulbecco's Modified Eagle Medium (DMEM), supplemented with 10% fetal bovine serum (FBS) and 1% penicillin-streptomycin. The cells were maintained in a humidified incubator at 37 °C with 5% CO₂. Upon reaching 80-90% confluence, the cells were subcultured using 0.25% trypsin-EDTA solution for digestion and passaging. All experiments were performed with cells in the logarithmic growth phase.

### 2.11. Transfection

The siRNAs targeting MUC16 (siRNA1, siRNA2, and siRNA3) as well as the non-targeting negative control siRNA were custom-designed based on the human MUC16 mRNA sequence and synthesized by a commercial supplier (GenePharma, Shanghai, China). All siRNAs were reconstituted and used according to the manufacturer's instructions.

Cells were seeded in 12-well plates and transfected at approximately 60% confluence. Different concentrations of target-specific siRNA were complexed with Lipofectamine 2000 reagent in Opti-MEM reduced serum medium according to the manufacturer's protocol (siRNA1: CAACUAUGGAUGUCACUAA siRNA2: GAUGCAACAUUCAUACCAA siRNA3: CUAGAUCCUCUGCGAUGAA). The siRNA-lipid complexes were added to the cells, which were then incubated for 6 hours before replacing the transfection mixture with complete growth medium. A non-targeting scrambled siRNA was used as a negative control (NC) to exclude off-target effects. The negative control siRNA sequence was: **5′-UUCUCCGAACGUGUCACGUTT-3′**, which does not show homology to any known human gene. All siRNAs were synthesized commercially and used according to the manufacturer's recommendations.

### 2.12. Quantitative Real-Time PCR (qRT-PCR)

Total RNA was extracted from transfected cells 48 hours post-transfection using TRIzol reagent, following the manufacturer's instructions. The purity and concentration of the RNA were measured spectrophotometrically. Subsequently, 1 μg of total RNA from each sample was reverse-transcribed into complementary DNA (cDNA) using a PrimeScript RT reagent kit. qPCR was then performed using SYBR Green Master Mix on a real-time PCR detection system. The specific primers for *MUC16* and the internal reference gene *GAPDH* were designed and utilized. The relative expression level of *MUC16* mRNA was calculated using the comparative 2^(-ΔΔCt) method, normalized to *GAPDH* and relative to the control group. Transfection efficiency was assessed indirectly by measuring MUC16 mRNA levels 48 h post-transfection using qRT-PCR. Among the three tested siRNAs, siRNA3 achieved the highest knockdown efficiency, resulting in approximately 72% reduction of MUC16 expression compared with the negative control group. This level of silencing indicates effective transfection and validates that the observed functional effects are attributable to MUC16 depletion. No significant cytotoxicity was observed in negative control siRNA-treated cells.

### 2.13. Wound Healing Assay

Following the designated transfection period, a standardized wound was created in the confluent cell monolayer using a sterile 200 μL pipette tip. The detached cells and debris were gently removed by washing the wells twice with phosphate-buffered saline (PBS). Fresh serum-free medium was then added to minimize cell proliferation. Images of the wound area were captured at 0 hours and 24 hours using an inverted phase-contrast microscope.

### 2.14. Transwell assay

After transfection, approximately 5×10⁴ cells in 200 μL of serum-free medium were seeded into the upper chamber. The lower chamber was filled with 500 μL of complete medium containing 10% FBS as a chemoattractant. The plates were incubated for 24 hours at 37 °C. Subsequently, non-migrated cells on the upper surface of the membrane were carefully removed with a cotton swab. The migrated cells on the lower surface were fixed with 4% paraformaldehyde, stained with 0.1% crystal violet, and photographed under a microscope.

### 2.15. Statistical Analysis

All analyses were conducted in R version 4.2.2. Parametric variables were compared by Student's t-test or ANOVA, and non-parametric data by Wilcoxon or Kruskal-Wallis tests as appropriate. Survival analyses were conducted using the log-rank test. A two-sided p-value < 0.05 was considered statistically significant.

## 3. Results

### 3.1. *MUC16* Overexpression Correlates with Poor Prognosis

**Figure 2:**
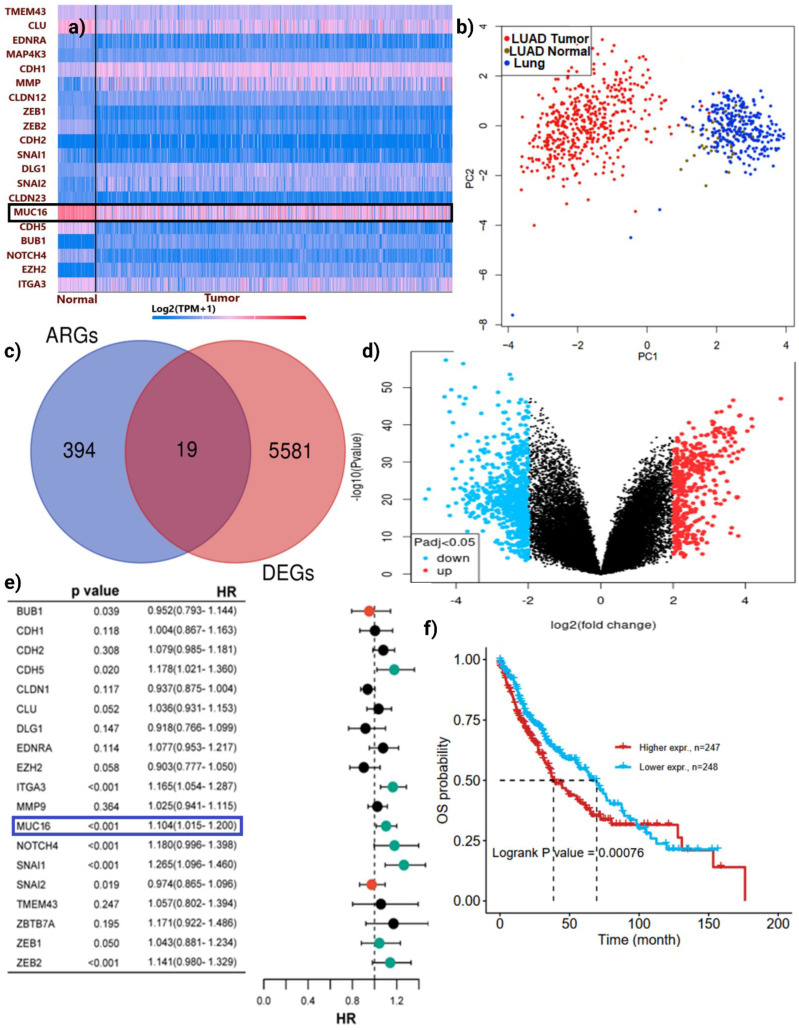
** Identification of *MUC16* as a novel anoikis-related gene associated with poor prognosis in LUAD: (a)** Heatmap showing the expression profiles of 19 overlapping ARDEGs between normal and LUAD tissues. The majority of ARGs were differentially expressed, with *MUC16* (highlighted) markedly upregulated in tumor samples. **(b)** PCA plot demonstrating clear separation between LUAD tumor (red) and normal lung (blue) samples, indicating distinct transcriptional patterns. **(c)** Venn diagram illustrating the intersection between 394 known ARGs and 5,581 LUAD differentially expressed genes (DEGs), identifying 19 common genes. **(d)** Volcano plot of DEGs (red = upregulated, blue = downregulated, Padj < 0.05) highlighting the distribution of significantly altered genes. **(e)** Forest plot of overlapping ARGs; *MUC16* (boxed) shows a strong association with poor overall survival (p < 0.001, HR = 1.04). **(f)** Kaplan-Meier survival curve comparing patients with high versus low *MUC16* expression levels in LUAD. Elevated *MUC16* expression correlates with significantly reduced overall survival (log-rank p = 0.00076).

To explore the molecular mechanisms underlying anoikis resistance in LUAD, we first performed transcriptomic profiling to identify anoikis-related differentially expressed genes (ARDEGs). A heatmap of the 19 overlapping ARDEGs demonstrated a marked distinction between normal and LUAD tissues, with several genes including *MUC16, CDH2, EZH2,* and *NOTCH4* showing substantial upregulation in tumor samples (**Figure 2a**). PCA revealed a distinct separation between tumor and normal samples, indicating clear transcriptional differences (**Figure 2b**). Differential expression analysis identified a total of 5,600+ DEGs, among which 19 genes overlapped with the curated anoikis-related genes (ARGs) set (**Figure 2c-d**). Notably, *MUC16* expression was consistently elevated in tumor tissues compared with adjacent normal lung samples, indicating that its upregulation may enhance adhesion-linked survival signaling and protect LUAD cells from detachment-induced apoptosis.

Univariate Cox regression analysis was conducted to assess the prognostic significance of these ARGs in LUAD (**Figure 2e**). Among the identified candidates, *MUC16* showed a highly significant association with poor overall survival (p < 0.001) and a hazard ratio (HR) of 1.04 (95% CI = 1.015-1.208), suggesting a potential role as a tumor-promoting factor. Other key regulators such as *NOTCH4* and *ZEB1/2* also demonstrated prognostic relevance; however, *MUC16* stood out as a top candidate due to its dual significance being both upregulated in tumors and strongly correlated with worse prognosis.

To determine the prognostic significance of *MUC16* expression in LUAD, a multivariate Cox proportional hazards model was constructed incorporating clinical and molecular covariates (Age, Gender, Race, Stage, and Tumor Purity). Among 421 evaluable patients (150 deaths), *MUC16* expression was significantly associated with overall survival (HR = 1.09, 95% CI = 1.01-1.18, p = 0.036), indicating that each unit increase in *MUC16* expression confers an approximately 9% higher risk of death.

As expected, advanced tumor stage was strongly associated with reduced survival, with Stage II (HR = 2.31, p < 0.001), Stage III (HR = 2.51, p < 0.001), and Stage IV (HR = 2.82, p = 0.002) showing progressively higher hazard ratios relative to Stage I. Tumor purity also emerged as a significant covariate (HR = 2.13, p = 0.031), suggesting that higher tumor cellularity is linked with poorer outcomes. Other variables, including age, gender, and race, did not reach statistical significance. To evaluate potential confounding effects, tumor purity distributions were compared between high and low MUC16 expression groups. No significant difference in tumor purity was observed between the two groups (Wilcoxon test, p > 0.05), indicating that the prognostic association of MUC16 expression is independent of tumor cellularity and not driven by differential stromal or immune content.

Model performance metrics (likelihood ratio test p = 3.73 × 10⁻⁷; Wald p = 6.15 × 10⁻⁶; log-rank p = 6.59 × 10⁻⁷) confirmed robust prognostic separation. Survival analysis further confirmed that LUAD patients with high *MUC16* expression had significantly reduced overall survival compared with those with low expression levels (log-rank p = 0.00076; **Figure 2f**). These findings identify *MUC16* as an independent adverse prognostic biomarker in LUAD, supporting a mechanistic model where *MUC16*-driven adhesion and survival signaling promote tumor progression by enabling cells to evade detachment-induced death, sustain metastatic competence tumor progression and anoikis resistance.

### 3.2. Functional Enrichment Analyses Reveal MUC16 Is Associated with Cell Adhesion and Survival Cascades

**Figure 3:**
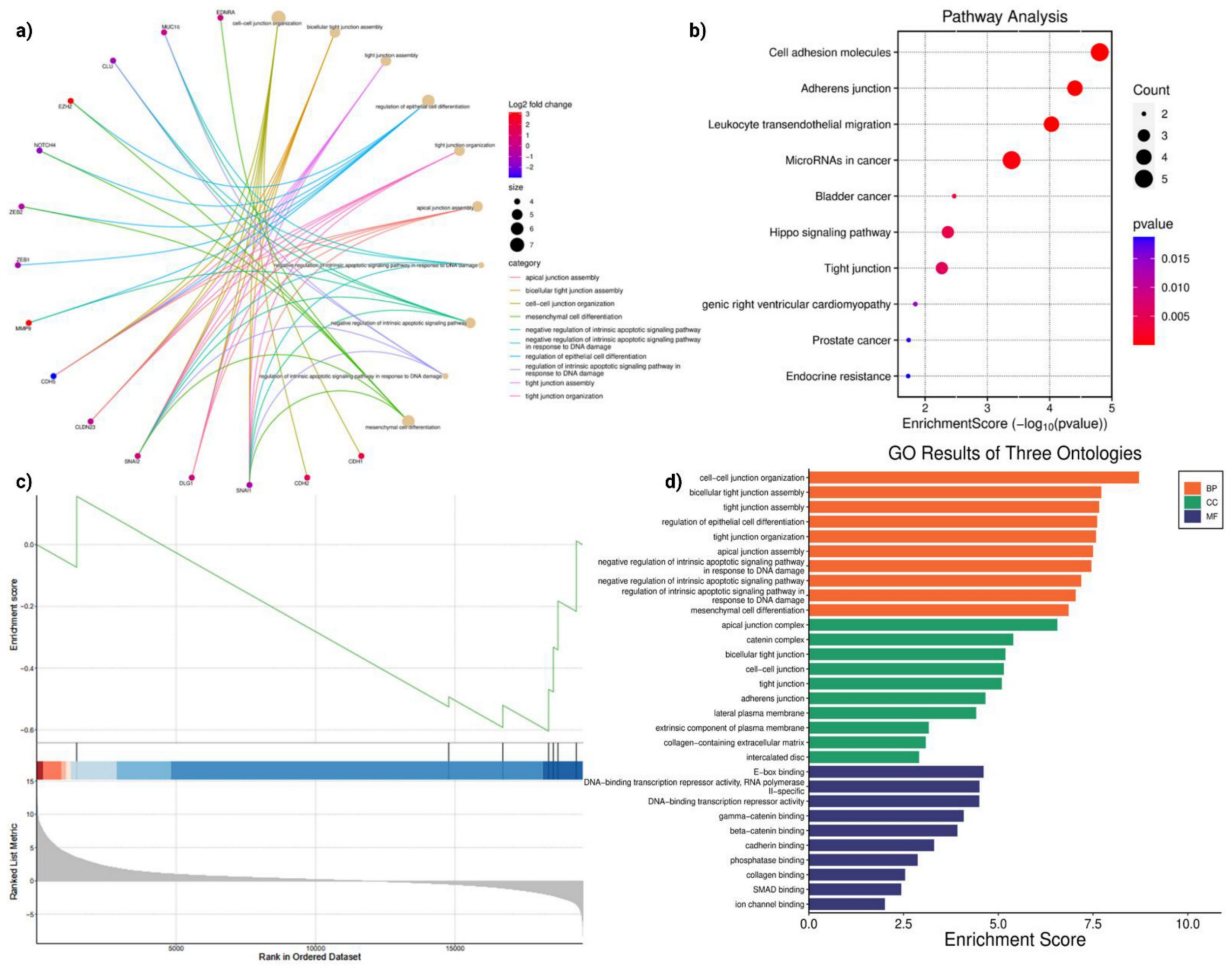
** Functional enrichment analyses reveal adhesion- and junction-related transcriptomic remodeling upon *MUC16* silencing in LUAD. (a)** Network visualization of differentially expressed anoikis-related genes showing enrichment in biological processes such as *cell-cell junction organization, tight junction assembly,* and *epithelial differentiation*. Node color represents log₂ fold change, and node size indicates significance of enrichment. **(b)** KEGG pathway analysis highlighting significant enrichment of *cell adhesion molecules*, *adherens junction*, *tight junction*, and *Hippo signaling* pathways, indicating that MUC16 regulates adhesion-dependent survival cascades. **(c)** GSVA demonstrating downregulation of adhesion-linked transcriptional programs and negative enrichment of DNA-binding transcriptional repressors, suggesting loss of pro-survival signaling upon *MUC16* silencing. GSVA highlights significant downregulation of EMT, focal adhesion, PI3K-AKT-mTOR signaling, apical junction, and actin cytoskeleton pathways upon MUC16 silencing (FDR < 0.05). **(d)** GO enrichment across three ontologies—BP, CC, and MF—showing predominant enrichment of junctional organization, plasma membrane complexes, and cadherin/catenin-binding functions.

Differential expression analysis of anoikis-related genes in LUAD revealed extensive transcriptional reprogramming following *MUC16* silencing. The interaction network (**Figure 3a**) highlighted multiple up- and down-regulated genes converging on biological processes linked to cell-cell junction assembly, tight junction organization, and epithelial differentiation. Genes such as *CDH1, CLDN3, CTNNB1,* and *JUP* were central to this network, indicating a coordinated alteration in the cell adhesion machinery. The enrichment of negative regulation of intrinsic apoptotic signaling further suggested a strong connection between adhesion loss and apoptotic susceptibility. These results show coordinated changes in genes involved in junction assembly, cytoskeletal organization, and apoptosis-related processes.

Pathway analysis (**Figure 3b**) demonstrated significant enrichment of cell adhesion molecules, adherens junction, and tight junction pathways, all critical regulators of anoikis. In addition, Hippo signaling, microRNAs in cancer, and endocrine resistance pathways were enriched, suggesting cross-talk between *MUC16* and canonical pro-survival cascades. The activation of Hippo signaling and junctional pathways is consistent with *MUC16'*s role in sustaining cytoskeletal integrity and suppressing detachment-induced apoptosis. Conversely, *MUC16* silencing disrupts junctional integrity and attenuates adhesion-mediated *FAK/PI3K-AKT* signaling, thereby restoring apoptotic susceptibility and impairing anchorage-dependent growth.

GSVA analysis (**Figure 3c**) ranked genes according to their enrichment in *MUC16-*silenced versus control LUAD cells. The negative enrichment score observed for DNA-binding transcription repressors and junctional regulators reflects a global suppression of transcriptional programs that promote anoikis resistance. This trend indicates that loss of *MUC16* represses transcriptional programs sustaining adhesion and survival, thereby reactivating apoptotic pathways, and consistent with reduced activity of adhesion- and survival-associated transcriptional programs. GO analysis (**Figure 3d**) across three ontologies (Biological Processes, Cellular Components, Molecular Functions) provided further insight into the mechanisms affected by *MUC16*.

GSVA analysis further revealed a coordinated downregulation of adhesion- and survival-associated transcriptional programs following MUC16 silencing. Among the most significantly suppressed pathways were Epithelial-Mesenchymal Transition (EMT) (normalized enrichment score [NES] = -1.87, FDR < 0.01), Focal Adhesion (NES = -1.72, FDR < 0.01), PI3K-AKT-mTOR signaling (NES = -1.65, FDR = 0.02), Apical Junction (NES = -1.58, FDR = 0.03), and Actin Cytoskeleton Organization (NES = -1.54, FDR = 0.04). These pathways are centrally involved in cell-matrix adhesion, cytoskeletal remodeling, and anchorage-dependent survival, supporting a role for MUC16 in sustaining adhesion-linked anoikis resistance.

Although pathway enrichment analyses consistently implicate FAK/PI3K-AKT signaling downstream of MUC16, the present data support an indirect regulatory relationship, whereby MUC16 influences adhesion- and cytoskeleton-associated transcriptional programs that converge on FAK-AKT pathway activation, rather than a direct protein-protein interaction.

### 3.3. MUC16 Occupies a Central Regulator in Aggressive LUAD Subtypes

**Figure 4:**
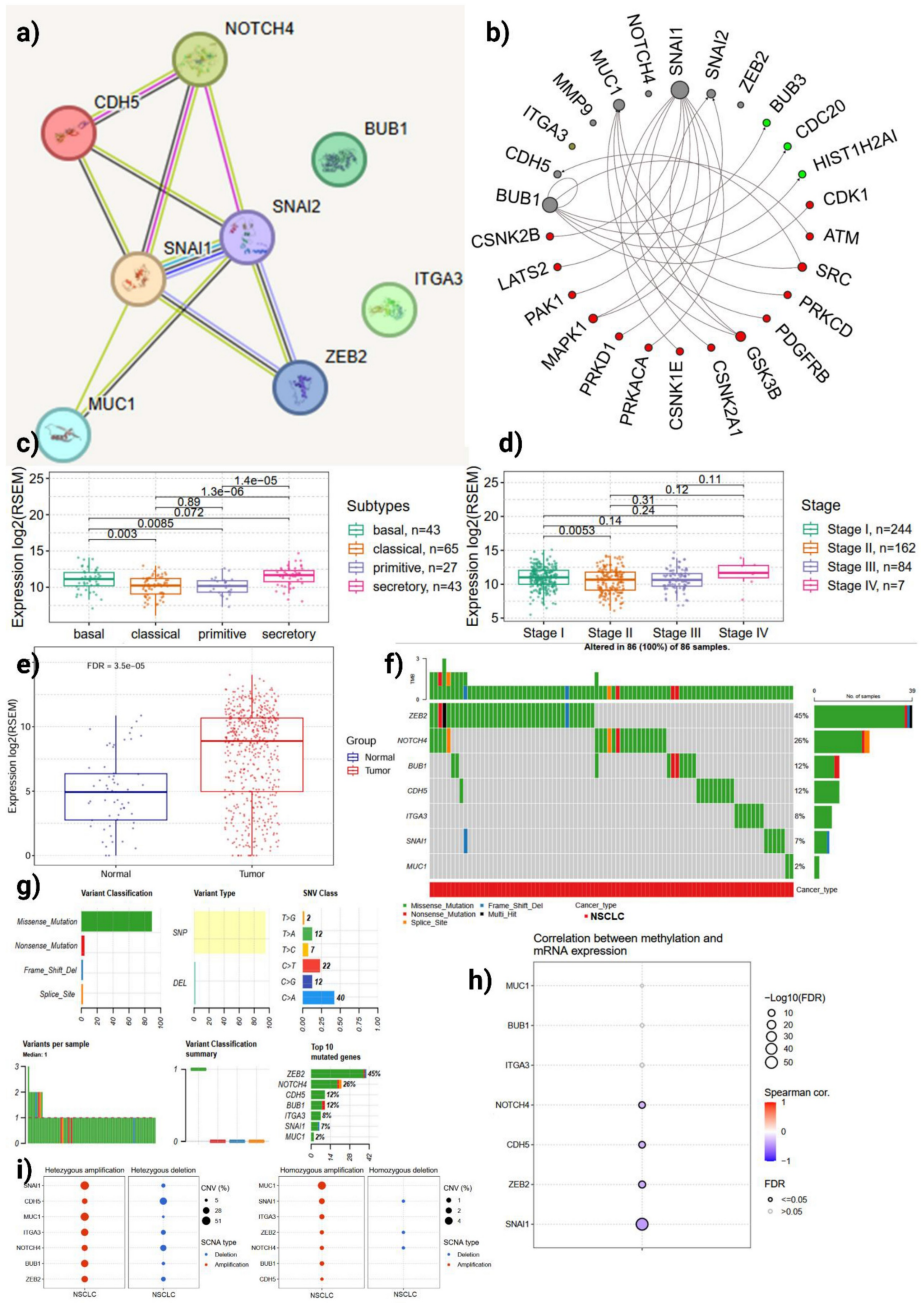
** Multi-omic characterization of *MUC16* in LUAD. (a)** Protein-protein and kinase interaction networks highlighting *MUC16* and its connectivity with *EMT* (*SNAI1/2, ZEB2, CDH5*) and cell-cycle kinases (*BUB1, CDK1*). **(b)** Boxplots showing elevated *MUC16* expression across LUAD molecular subtypes, pathological stages, and tumor versus normal tissues (p-values shown) **(c-e)** Boxplots of *MUC16* expression across transcriptomic subtypes, pathologic stage, and tumor vs. normal (statistical annotations as shown). **(f-g)** Oncoplot and SNV and mutation spectra illustrating recurrent *MUC16* copy-number gains and missense variants co-occurring with EMT-related alterations. **(h)** Correlation between DNA methylation and mRNA expression showing promoter hypomethylation-associated *MUC16* activation. **(i)** Copy-number variation showing recurrent amplifications consistent with increased transcriptional upregulation *MUC16* expression.

Network analysis of the anoikis-related DEGs placed *MUC16* within a tightly connected module containing canonical EMT regulators (*SNAI1, SNAI2, ZEB2, CDH5*), the Notch family member *NOTCH4*, and cell-cycle/mitotic checkpoint nodes (*BUB1, CDK1*) (**Figure 4a-b**). Edges connecting *MUC16* to EMT transcription factors and to kinases linked to cell-cycle progression indicate that *MUC16* is biochemically and functionally poised to coordinate both adhesive/EMT programs and proliferation signals. *MUC16* occupies a central regulatory position within the anoikis-EMT-cell-cycle interaction module, suggesting that it coordinates epithelial plasticity and proliferation signaling to maintain detachment survival and drive metastatic progression.

Comparative expression analyses show that *MUC16* expression is significantly higher in the LUAD transcriptional subtypes associated with aggressive biology (statistically significant differences across subtypes; pairwise p-values shown, 1.3×10⁻⁶ and 1.4×10⁻⁵ between selected groups) (**Figure 4c**). Similarly, *MUC16* expression increases with pathologic stage (stage comparisons annotated in **Figure 4d**), and tumor samples display robust upregulation relative to normal lung (tumor vs. normal, FDR = 3.5×10⁻⁵) (**Figure 4e**). These data collectively indicate that *MUC16* overexpression enhances cellular adaptability to detachment stress, providing a selective advantage in aggressive LUAD subtypes characterized by high metastatic potential.

Oncoplot and mutation-summarizing plots show heterogeneous alteration patterns across the ARDEGs (**Figure 4f-g**). While SNV (predominantly missense changes) occur across multiple genes, structural and copy-number events represent a major source of *MUC16* dysregulation in LUAD: *MUC16* alterations frequently co-occur with changes in EMT and integrin genes (*ITGA3, CDH5*) and with the Notch/mitotic axis (*NOTCH4, BUB1*). The low overall burden of disruptive SNVs in key EMT regulators, together with recurrent copy-number gains and expression upregulation, suggests that *MUC16* amplification and transcriptional activation act as gain-of-function mechanisms that amplify adhesion and survival signaling, thereby enhancing anoikis resistance and metastatic potential in LUAD.

Correlation analysis between DNA methylation and mRNA levels identifies a significant inverse relationship for *MUC16* (FDR-adjusted; Spearman correlation shown in **Figure 4h**), consistent with promoter or regulatory hypomethylation contributing to raised transcript abundance. The MUC16 promoter region with CpG islands and transcription start site (TSS) annotations were illustrated in **Supporting Figure S1**. This epigenetic signature supports a model in which hypomethylation facilitates *MUC16* overexpression in tumor cells, augmenting downstream EMT/integrin and pro-survival signaling. This epigenetic deregulation reinforces a gain-of-function model wherein hypomethylation-induced *MUC16* overexpression activates adhesion-linked pro-survival signaling cascades, promoting detachment tolerance in LUAD.

Single copy-number analysis and SCNA summaries indicate recurrent focal or arm-level amplifications for a subset of ARDEGs, including *MUC16*, that are accompanied by increased mRNA expression (**Figure 4i**). The concordance between copy-number gain and transcriptional upregulation reinforces *MUC16*'s role as an activated effector in LUAD biology rather than a passenger gene. These structural amplifications likely sustain continuous activation of *MUC16*-mediated *FAK-AKT* signaling, providing LUAD cells with enhanced anchorage-independent survival and metastatic capacity.

### 3.4. MUC16 Alterations Remodel the Immune Landscape and Drug Sensitivity

**Figure 5:**
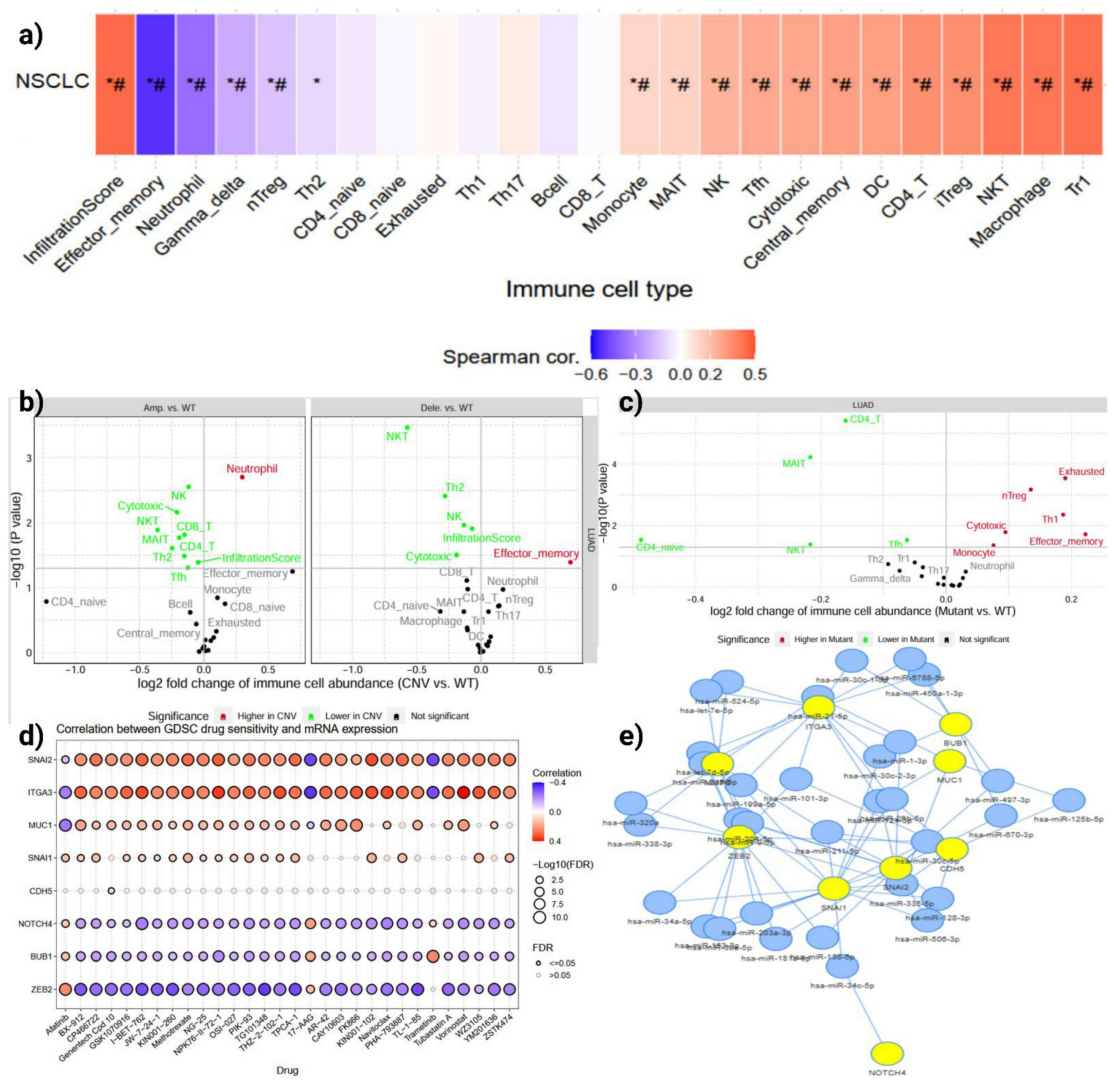
** Immune, pharmacogenomic, and miRNA correlates of *MUC16* activation in LUAD. (a)** Correlation heatmap showing associations between *MUC16* expression and immune-cell infiltration scores across LUAD samples. **(b, c)** Comparison of immune-cell abundance between copy-number altered, mutant, and wild-type MUC16 tumors; amplification or mutation reduces cytotoxic infiltration while enriching suppressive cell types. **(d)** Correlation between GDSC drug sensitivity and *MUC16* mRNA levels demonstrating resistance to cytotoxic agents and selective vulnerability to kinase inhibitors. **(e)** miRNA-mRNA interaction network connecting *MUC16* with EMT and integrin-signaling partners via shared miRNAs (miR-34a-5p, miR-200b-3p).

Immune deconvolution of LUAD transcriptomes revealed significant correlations between *MUC16* expression and immune infiltration patterns (**Figure 5a**). High *MUC16* levels were inversely correlated with infiltration of cytotoxic and memory T cells (CD8⁺, CD4⁺_naïve, effector-memory subsets), while positively associated with macrophage and exhausted T-cell fractions. This pattern indicates that *MUC16* overexpression coincides with reduced anti-tumor effector activity and enrichment of immunoregulatory cell populations.

Copy-number and mutation-stratified analyses reinforced this observation (**Figure 5b, c**): *MUC16* amplification or mutation was accompanied by a marked decline in CD8⁺ and NK-cell abundance and a rise in Th2, Treg, and exhausted T-cell signatures. These findings suggest that *MUC16* activation promotes an immune-excluded or suppressive microenvironment, potentially protecting detached LUAD cells from immune-mediated clearance during anoikis escape. Mechanistically, this immune exclusion pattern suggests that *MUC16*-mediated adhesion and survival signaling may also facilitate immune evasion, shielding detached LUAD cells from NK- and T-cell-mediated apoptosis during metastatic dissemination.

Integration of LUAD gene-expression profiles with the Genomics of Drug Sensitivity in Cancer (GDSC) dataset demonstrated that *MUC16*-high tumors exhibit distinctive pharmacogenomic behavior (**Figure 5d**). *MUC16* expression correlated positively with resistance to microtubule-targeting and DNA-damaging agents but inversely with sensitivity to PI3K, mTOR, and kinase-inhibitory compounds. These changes were accompanied by enrichment of apoptosis-related gene sets.

Network modeling of *MUC16*-associated miRNAs revealed a dense regulatory cluster linking *MUC16, NOTCH4, SNAI1/2, ITGA3, and BUB1* through shared miRNAs such as *hsa-miR-34a-5p*, *hsa-miR-200b-3p*, and *hsa-miR-429* (**Figure 5e**). These miRNAs are known suppressors of EMT and integrin signaling; their coordinated downregulation in LUAD may permit *MUC16* overexpression and maintenance of the mesenchymal, anoikis-resistant state. Thus, dysregulation of the *MUC16*-miRNA axis likely underlies simultaneous activation of EMT and survival pathways, further reinforcing immune evasion and metastatic competency.

### 3.5. MUC16 Knockdown Suppresses A549 Cell Migration and Invasion

**Figure 6:**
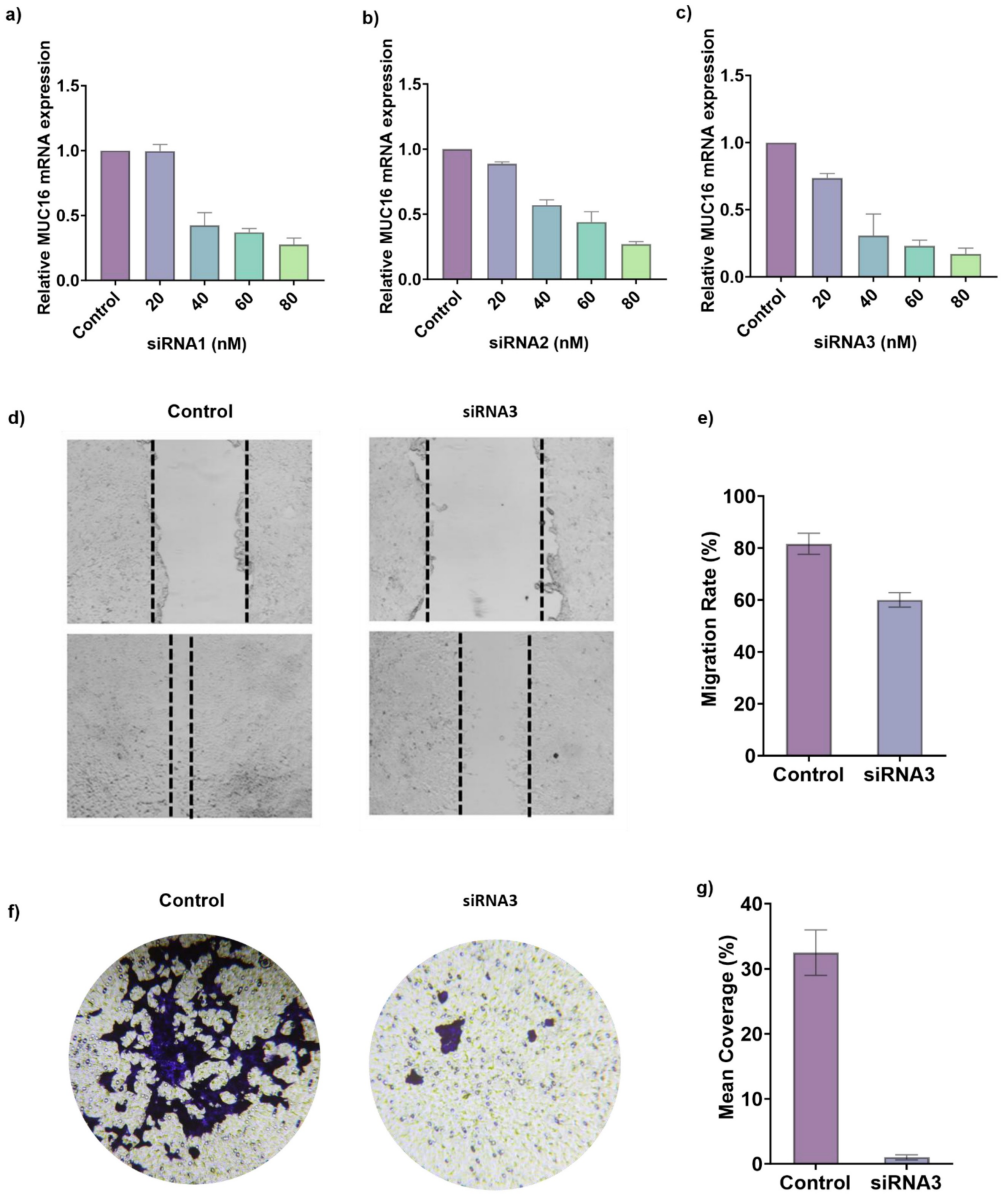
** Experimental validation of *MUC16* knockdown via siRNA in LUAD cells. (a-c)** Quantitative RT-PCR analysis showing relative *MUC16* mRNA expression levels following transfection with increasing concentrations (20-80 nM) of three distinct siRNAs (siRNA1, siRNA2, and siRNA3). *MUC16* expression decreased in a dose-dependent manner compared with the control group, confirming efficient gene silencing. Data are presented as mean ± SD (n = 3); p < 0.05, one-way ANOVA. **(d, e)** Representative images of wound-healing assays showing reduced migratory capacity of LUAD cells upon *MUC16* silencing. Cells transfected with siRNA3 exhibited markedly delayed wound closure compared with the control and negative control (NC) groups at 0 h and 24 h post-scratch. (**e**) Wound closure was quantified as the percentage of gap closure relative to baseline. Data are presented as mean ± SD from three independent experiments. Control cells achieved 95.6 ± 4.3% wound closure at 24 h, whereas MUC16-silenced cells exhibited significantly reduced closure (41.2 ± 3.8%, p < 0.001). **(f)** Transwell invasion assays demonstrating that *MUC16* knockdown significantly inhibits invasive potential in LUAD cells. Fewer cells penetrated the Matrigel-coated membranes following siRNA3 transfection relative to control. (**g**) Qualification of the transwell invasion assays. Scale bars, 100 μm.

To experimentally validate these computational insights, siRNA-mediated silencing of *MUC16* were performed. Firstly, to determine the silencing efficiency of *MUC16,* A549 cells were transfected with three distinct siRNAs (siRNA1, siRNA2, and siRNA3) at various concentration (20, 40, 60, 80 nM). Quantitative real-time PCR (qRT-PCR) results revealed a significant dose-dependent downregulation of *MUC16* mRNA expression in all siRNA-treated groups compared to the negative control (**Figure 6a-c**). Among the tested sequences, siRNA3 exhibited the strongest inhibitory effect, achieving approximately 72% suppression of *MUC16* expression relative to the control group (p < 0.001), while siRNA1 and siRNA2 reduced expression by ~58% and ~49%, respectively. These results confirm that siRNA3 effectively silenced *MUC16* expression and was therefore selected for subsequent functional assays.

To further evaluate the effect of *MUC16* silencing on cell motility, a wound healing assay was performed following siRNA transfection. As shown in **Figure 6d**, control A549 cells exhibited a pronounced reduction in wound width after 24 hours, indicating rapid migration of cells into the scratched area. In contrast, *MUC16*-silenced cells demonstrated a markedly delayed wound closure, with the scratch area remaining significantly wider than that of the control or NC group over the same time period. Quantitative analysis of the wound healing assay demonstrated that control A549 cells exhibited a wound closure rate of 95.6 ± 4.3% at 24 h. In contrast, MUC16-silenced cells showed a significantly reduced migratory capacity, with closure rates of 41.2 ± 3.8% in the siRNA3 group, indicating that MUC16 knockdown markedly impairs cell migration (**Figure 6e,** p < 0.001). This substantial decrease in migration rate indicates that *MUC16* plays a crucial role in promoting A549 cell motility. The observed impairment in wound healing following *MUC16* knockdown suggests that the gene facilitates cytoskeletal reorganization and lamellipodia extension key processes involved in directional cell migration.

In addition, to elucidate the role of *MUC16* in regulating lung cancer cell motility, transwell migration assays were performed following siRNA3-mediated knockdown. The transwell migration assay demonstrated a pronounced decrease in the number of migrated cells following *MUC16* silencing (**Figure 6f**). Crystal violet staining revealed densely populated clusters on the lower membrane surface in control wells, whereas siRNA-treated cells showed substantially fewer and smaller clusters, indicating reduced migration through the transwell membrane. Quantitative analysis showed a 65-70% decrease in migrated cells in the siRNA group relative to the control (**Figure 6g,** p < 0.001). Together, the wound-healing and transwell assays confirm that *MUC16* functionally promotes adhesion-dependent survival and motility, providing ediences of its role in protecting LUAD cells from detachment-induced apoptosis and enhancing metastatic behavior.

## 4. Discussion

Our study demonstrates that *MUC16* silencing disrupts multi-level adhesion networks and survival pathways, thereby re-sensitizing LUAD cells to anoikis. Integration of KEGG, GO, and GSVA analyses supports a mechanistic model in which *MUC16* promotes anoikis resistance by maintaining junctional integrity and activating adhesion-dependent survival signaling. Its silencing leads to disassembly of adherens and tight junction complexes, down-regulation of Hippo and catenin-associated pathways, and activation of apoptotic regulators. Consequently, LUAD cells become unable to sustain growth under detachment, implicating MUC16 as a central regulator of adhesion-mediated survival and a potential therapeutic target to overcome anoikis resistance. These transcriptomic, multi-omic, and functional findings establish a mechanistic framework in which *MUC16* integrates adhesion and survival signaling to maintain anoikis resistance and metastatic competence in lung adenocarcinoma.

Metastatic dissemination of LUAD critically depends on tumor cells' ability to resist anoikis. In this study, we identified *MUC16* as a novel mediator of anoikis resistance through an integrated bioinformatics and experimental validation approach. Our findings reveal that *MUC16* promotes LUAD cell survival by sustaining cell-cell junction integrity and activating adhesion-linked survival signaling, thereby facilitating metastatic potential. Among nineteen ARDEGs, *MUC16* emerged as one of the most significantly upregulated genes in LUAD tissues compared with normal lung. Although *MUC16* has been widely characterized as *CA125* in ovarian malignancies, its biological role in lung cancer has remained largely unexplored. Our study provides the first direct evidence that *MUC16* confers anoikis resistance in LUAD, positioning it as a mechanistic determinant of metastatic fitness. Li et al. demonstrated that *MUC16* knockdown in lung cancer cell lines reduced proliferation, migration and in vivo growth via the *JAK2/STAT3/GR/TSPYL5* axis 24, 25. Other studies show frequent mutation and over-expression of *MUC16* in lung adenocarcinoma 26, 27 and its signature as a poor prognostic marker when co-expressed with other mucins 28, 29. However, none of these prior works have addressed the explicit mechanism of anoikis (detachment-induced apoptosis) in lung cancer in the context of *MUC16* 30. Thus our work provides the first evidence (to our knowledge) that *MUC16* facilitates anoikis resistance in LUAD a key step in metastatic dissemination when cells detach from the primary site and survive in circulation or in new microenvironments.

GO and pathway enrichment analyses revealed that *MUC16* were strongly enriched in biological processes involving cell-cell junction organization, tight junction assembly, regulation of epithelial cell differentiation, and negative regulation of apoptosis. Cellular component terms such as apical junction complex, adherens junction, and collagen-containing extracellular matrix further pointed toward *MUC16*'s involvement in maintaining epithelial integrity and anchorage-dependent survival. Molecular function terms including β-catenin binding, cadherin binding, and phosphatase binding suggest that *MUC16* interacts with adhesion and cytoskeletal regulators that suppress apoptotic signaling following detachment. These results align with the hypothesis that *MUC16* enhances cell adhesion and activates pro-survival signaling pathways downstream of *FAK-PI3K-AKT* and β-catenin, thereby preventing anoikis. Previous studies have shown that FAK activation promotes integrin clustering and cell spreading, supporting cell viability during loss of matrix contact 31, 32. Our findings extend this paradigm by identifying *MUC16* as an upstream regulator of this adhesion-dependent survival circuitry. Jonckheere et al. linked *MUC16* (via a *MUC4/MUC16/MUC20* signature) with genes involved in cell adhesion and cell-cell junctions 28, 33. Additionally, previous work in other cancer types supports the ability of *MUC16* to enhance metastasis via signaling pathways: in pancreatic ductal adenocarcinoma (PDAC), *MUC16* drives liver metastasis by up-regulating Neuropilin-2 (NRP2) via JAK2/STAT1 signalling 34, 35. In triple-negative breast cancer, MUC16 promotes lung metastasis by modulating the RNA-binding protein ELAVL1 (HuR) and thereby influencing post-transcriptional regulation of metastasis-associated genes 28, 36, 37. These data support the paradigm of *MUC16* acting beyond a biomarker to a functional promoter of metastasis. Our findings extend this paradigm by pinpointing the survival of detached tumor cells (anoikis resistance) as a mechanistic axis in LUAD.

The Kaplan-Meier survival analysis in our study (log-rank p = 0.00076) confirms that higher *MUC16* expression marks worse overall survival in LUAD. Our univariate Cox regression (HR = 1.04, 95 % CI = 1.015-1.208) further corroborates its prognostic significance. This aligns with reports that MUC16 mutation status associates with tumor mutation burden (TMB) and immunotherapy response in LUAD: Liu et al. developed a MUC16 mutation-associated immune prognostic model showing that *MUC16*^mut frequent status corresponded with higher TMB and improved survival 38. The fact that MUC16 may modulate both cell survival (via anoikis resistance) and immune escape (through NK-cell inhibition, Siglec-9 interactions) underscores its multifaceted role in tumor biology. An apparent complexity arises when considering previous reports linking MUC16 mutation to higher tumor mutation burden (TMB) and improved immunotherapy response in LUAD, as described by Liu et al. In contrast, our findings demonstrate that high MUC16 expression is associated with reduced cytotoxic immune-cell infiltration and an immunosuppressive tumor microenvironment 39, 40. This distinction likely reflects fundamentally different biological consequences of mutational disruption versus transcriptional overexpression 39. While MUC16 mutations may increase neoantigen generation and enhance tumor immunogenicity, elevated MUC16 expression may represent a gain-of-function state that promotes immune evasion through physical shielding 41, 42, altered adhesion signaling, and suppression of effective antitumor immune surveillance 43, 44. Together, these observations underscore the context-dependent roles of MUC16 in modulating tumor-immune interactions in lung adenocarcinoma.

Indeed, in lung cancer, elevated serum *CA125* (*MUC16* antigen) often marks advanced disease and metastasis (including pleural effusion) 45. It is important to clarify the nature of the relationship between MUC16 and the FAK/PI3K-AKT signaling axis. While our enrichment, GSVA, and network analyses consistently highlight activation of FAK-AKT-related pathways in association with elevated MUC16 expression, the present study does not provide evidence for a direct physical interaction between MUC16 and FAK or PI3K components. Instead, our findings support a model in which MUC16 indirectly modulates FAK/PI3K-AKT signaling through transcriptional and functional regulation of adhesion molecules, integrins, and cytoskeletal regulators, thereby sustaining anchorage-dependent survival signaling 36.

This interpretation is supported by prior studies in other cancer types 46. In pancreatic ductal adenocarcinoma, MUC16 has been shown to promote metastatic behavior through regulation of adhesion-associated signaling networks involving Neuropilin-2 and downstream survival pathways 47. Similarly, in triple-negative breast cancer, MUC16 enhances metastatic progression by modulating RNA-binding proteins and post-transcriptional programs that ultimately converge on pro-survival and cytoskeletal signaling cascades 9, 48. Together, these studies suggest that MUC16 functions as an upstream coordinator of adhesion-linked survival signaling rather than a direct kinase-interacting protein. In lung adenocarcinoma, our integrative analyses extend this paradigm by identifying MUC16-driven transcriptional remodeling of adhesion and junctional programs as a key mechanism linking MUC16 overexpression to FAK/PI3K-AKT activation and anoikis resistance.

Functionally, *MUC16* knockdown markedly suppressed migratory and invasive capacities, as demonstrated by wound healing and transwell assays. These findings support the hypothesis that *MUC16* facilitates cytoskeletal remodeling and cell motility, two hallmarks of anoikis-resistant metastatic cells. The observed reduction in migratory ability upon silencing also suggests disruption of *MUC16*-integrin/FAK signaling, leading to diminished activation of downstream AKT survival pathways. Together, these results substantiate a mechanistic model where *MUC16* preserves cell-cell junction integrity and adhesion signaling to prevent anoikis and promote metastatic competence. Although migration and invasion assays do not directly measure anoikis, they provide important functional readouts of adhesion-dependent survival mechanisms. Efficient cell migration and transwell invasion require dynamic regulation of cell-matrix adhesion, cytoskeletal reorganization, and activation of pro-survival signaling pathways that overlap substantially with those governing anoikis resistance 49. Cells capable of sustaining motility under reduced or transient attachment conditions typically exhibit enhanced tolerance to detachment-induced stress, a defining feature of anoikis-resistant tumor cells. Nevertheless, we acknowledge that the present study did not include direct anoikis-specific apoptosis assays, such as suspension culture-induced apoptosis, annexin V/propidium iodide staining, or caspase-3/7 activation under non-adherent conditions 50. Future studies incorporating these assays will be valuable to directly quantify detachment-induced apoptosis and further substantiate the mechanistic role of MUC16 in regulating anoikis resistance in lung adenocarcinoma.

From a therapeutic perspective, targeting *MUC16* directly or through its downstream FAK-AKT signaling axis may restore anoikis sensitivity and inhibit metastatic progression. Indeed, FAK inhibitors and PI3K-AKT blockers are already in clinical testing for solid tumors, suggesting that *MUC16* expression could serve as a predictive biomarker for responsiveness to adhesion-signaling inhibitors. Furthermore, antibody-based or siRNA-based *MUC16* suppression strategies, as previously applied in ovarian cancer models, may offer promising translational avenues for LUAD management.

Despite the compelling findings, several limitations warrant consideration. Firstly, although our siRNA experiments clearly demonstrate the role of *MUC16* in promoting detachment survival and metastatic behavior *in vitro*, in vivo validation remains essential. Secondly, the downstream signaling mechanisms through which *MUC16* regulates adhesion and survival require further elucidation. While our enrichment analyses implicate FAK, integrins, cadherins, and ECM components, direct mechanistic evidence remains to be established. Thirdly, although survival analyses across TCGA cohorts revealed strong prognostic associations, validation in independent patient populations is still required. Immunohistochemical staining of *MUC16* on LUAD tissue microarrays and correlation with detachment or apoptosis markers (cleaved caspase-3, E-cadherin loss) would reinforce the clinical relevance of our findings.

Despite the strong integrative and functional evidence presented, several limitations should be acknowledged. First, although our in vitro data demonstrate that MUC16 promotes adhesion-dependent survival, migration, and invasion, in vivo validation remains necessary to fully establish its role in metastatic dissemination. Future studies could employ tail vein injection or orthotopic lung implantation models using MUC16-silenced LUAD cells to directly assess metastatic colonization, lung tumor burden, and survival of detached tumor cells in vivo. Such models would provide direct evidence linking MUC16-mediated anoikis resistance to metastatic fitness in a physiological process.

Second, functional validation in the present study was performed exclusively in A549 cells, a KRAS-mutant LUAD model. While this cell line is widely used to study lung cancer metastasis and adhesion signaling, LUAD is a heterogeneous disease. Therefore, extending these findings to additional LUAD cell lines with distinct molecular backgrounds, such as H1299 (TP53-null) or PC9 (EGFR-mutant) cells, would further strengthen the generalizability of our conclusions. Notably, the consistent association of MUC16 overexpression with poor prognosis, copy-number gain, epigenetic deregulation, and immune suppression across large patient cohorts supports a broader relevance of MUC16-driven anoikis resistance beyond a single cell model. These future directions will help refine the mechanistic and translational significance of MUC16 as a therapeutic target in lung adenocarcinoma.

## 5. Conclusion

Our work identifies *MUC16* as a novel mediator of anoikis resistance in lung adenocarcinoma, linking its upregulation with detached tumour-cell survival, metastatic potential, and a poor prognosis. By bridging bioinformatics and functional validation, we not only reinforce *MUC16*'s clinical relevance in LUAD but also suggest a new mechanistic axis of anoikis evasion that underpins its tumor-promoting role. These findings open new avenues for anti-metastasis therapeutic strategies targeting *MUC16* and its downstream adhesion-signalling network in lung cancer.

## Supplementary Material

Supplementary figure.

## Data Availability

The data are included in this manuscript and supporting information.
