# Supplementary material for: Targeting MUC16 to Reverse Anoikis Resistance: A Promising Strategy for Metastatic Lung Adenocarcinoma Therapy
Source: J Cancer. 2026 Mar 30;17(4):819–34. doi: 10.7150/jca.131494 (PMC13105147; doi:10.7150/jca.131494)
Supplement: Supplementary file 1 — Supplementary figure. [file jcav17p0819s1.pdf]

Supplementary Figure S1:

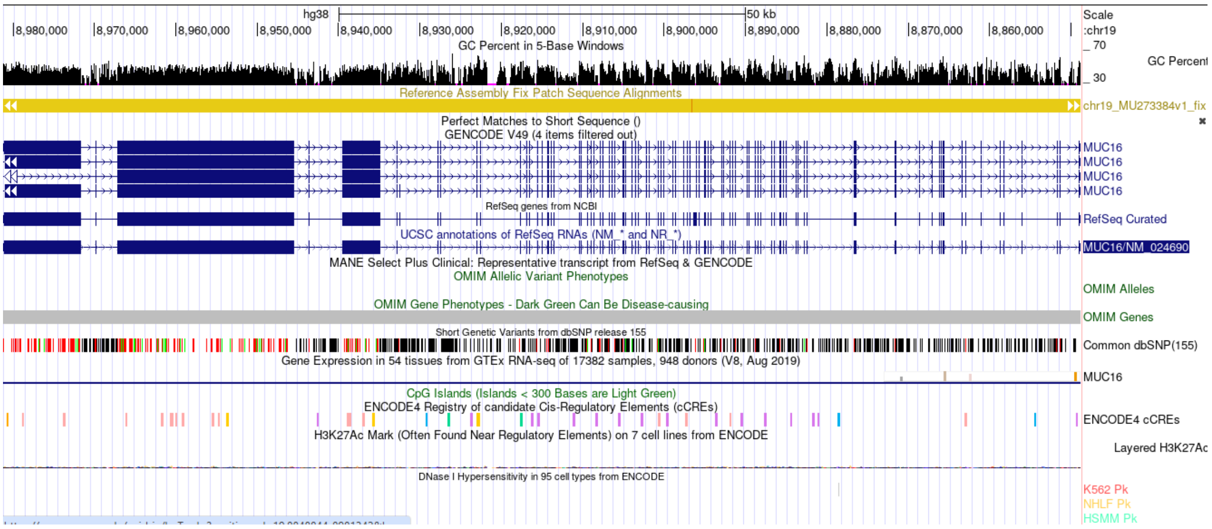

Figure S1: Promoter CpG sites correspond to Illumina 450K probes located within TSS200, TSS1500, 5′UTR, and first exon regions of MUC16.
